# The Mediating Role of Fatigue Between Mental Health and Its Associated Factors: Evidence From Chinese Healthcare Workers During the COVID-19 Pandemic

**DOI:** 10.3389/fpsyt.2021.665992

**Published:** 2021-06-09

**Authors:** Rong Peng, Wensu Zhou, Dexin Zhou, Muyang Chu, Li Ling

**Affiliations:** ^1^National Economics Research Center, Guangdong University of Finance and Economics, Guangzhou, China; ^2^Department of Medical Statistics, School of Public Health, Sun Yat-sen University, Guangzhou, China

**Keywords:** healthcare workers, COVID-19, mental health, fatigue, China

## Abstract

The aim of this study was to explore the mediating role of fatigue between mental health and its associated factors, including workload, social support, and occupational protection, among healthcare workers during the COVID-19 pandemic in China. A national cross-sectional survey was performed to collect data from healthcare workers who have attended to patients with COVID-19. Structural equation modeling (SEM) was conducted to test the mediating effect of fatigue. The results indicated that fatigue was a significant mediator of mental health. The proportion of indirect effect with regards to the total effect of workload on mental health was 54.2%, significantly greater than other factors such as social support (19.7%) and occupational protection (23.4%). The findings confirmed that workload, social support, and occupational protection both had indirect and direct effects on mental health status through fatigue. To some extent, potential interventions designed to alleviate fatigue would reduce mental health problems among healthcare workers during the COVID-19 pandemic.

## Introduction

The widespread outbreak of the 2019 coronavirus disease (COVID-19) has caused considerable impacts on people's psychobiological status (1–3). A review of the literature revealed that the COVID-19 pandemic was associated with adverse mental health consequences (4). Patients with COVID-19 and their families (3, 5), individuals with existing physical or psychiatric morbidities (6), and healthcare staff (2, 7, 8) were identified to be at higher risk of adverse mental health outcomes (4). Despite the fact that the importance of mental health care and psychological interventions has received widespread attention during the COVID-19 outbreak (6, 9, 10), intervention measures to cope with psychosocial challenges problems have been limited (11–14).

This study focused on healthcare workers, that is, the people who have treated or managed patients with COVID-19. These workers faced a higher risk of being infected and possibly suffered from a higher level of occupational stress during the outbreak of the COVID-19 pandemic. Several studies have addressed the impact of COVID-19 on the mental health of healthcare workers (8, 15, 16). Scholars found that healthcare workers suffered significant mental health problems during the COVID-19 outbreak (15). For example, medical workers were more likely to develop psychosocial problems than non-medical workers (8). Moreover, the front-line medical staff in close contact with infected patients were more likely to suffer anxiety and depression than administrative staff and the general public (16). The risk factors that could contribute to adverse mental health outcomes include long working hours, risk of infection, shortages of protective equipment, loneliness, physical fatigue, and separation from families (8, 15).

However, there is a lack of evidence in the literature regarding the mechanism of those risk factors on mental health problems among healthcare workers. Moreover, little attention has been paid to the mediating role of fatigue in the relationship between mental health and its associated factors. Fatigue is a biological symptom reflecting human body and mind (17) which generally result in less vigilance and declining cognitive functioning (18). Several dimensions including mental fatigue, physical fatigue, reduced activity, and reduced motivation were designed to reflect the complicated connotations of fatigue (19). Fatigue was a great challenge for healthcare workers, because cumulative fatigue would cause serious mistakes in jobs and threat to the safety of patients (20). Evidence suggests that fatigue has a significant impact on healthcare workers' mental health (21).

In addition, some investigators have evaluated the associations of fatigue with social support, workload, and occupational protection (20, 22, 23). High workload which accompanied by insufficient rest time was associated with fatigue. It was explained by cognitive-energetical theories of information processing (22). Social support is an effective source of support and a vital coping factor against difficulty, and it can help to relieve emotional and physical fatigue (23). During the COVID-19 pandemic, the shortage of medical protective equipment was one of the reasons for psychological distress (8, 24) which might further lead to mental fatigue or reduced activity (20). Based on knowledge of previous researches on mental health, fatigue and their risk factors, it was assumed that the effect of workload, social support and occupational protection on mental health problem might be mediated via fatigue.

The aim of this study was to examine the mediating role of fatigue between mental health and associated factors, including workload, social support and occupational protection, among healthcare workers during the COVID-19 epidemic in China. This study served as a national survey to identify the association between workload, social support, occupational protection and mental health and examined fatigue as a mediator. The findings from this research will help to better understand factors associated with mental health, and specifically the influencing mechanism of fatigue on the mental health of healthcare workers. These findings could have significant implications for effective interventions designed to improve healthcare workers' mental health and well-being.

## Methods

### Design and Participants

#### Survey Design

This was a cross-sectional study conducted through an online survey on the Wenjuanxing platform from February 27 to March 12, 2020 in China. The Wenjuanxing platform is an open, widely accepted and online system to collect information through individual WeChat account (the most common social media in China) and each IP address is only allowed to submit one questionnaire. Although it was limited by snowball sampling method, it was voluntary, anonymous and not restricted by regions. Besides, we advertised this investigation through public health school of Sun Yat-sen University and distributed the survey links through healthcare workers' WeChat which ensured representativeness of the sample.

The survey enrolled healthcare workers from 31 provinces in China. The survey period corresponded to a period of decline following that of the highest rate of infection of the COVID-19 epidemic outbreak in China; this represented a period when worker distress was expected to be high. The questionnaire included six sections: Socio-demographic information, workload, social support, occupational protection, fatigue, and mental health status.

#### Participants

A total of 2,077 healthcare workers attended the survey of China and provided full information, among them 16 persons did not work at least one full shift during the COVID-19 outbreak. After excluding the 16 persons mentioned above, 2,061 participants were included in this study.

### Ethics

The study protocol was approved by the Research Ethics Committee of Sun Yat-sen University, Guangzhou, China. Because the questionnaire was anonymous, we assumed that participants consented to participate in our study by returning the questionnaire.

### Variables and Measurement

#### Socio-Demographic Variable

Socio-demographic variables included gender (Male = 0, Female = 1), age, education level (Less education than a bachelor's degree = 0, Bachelor's degree = 1, Master's or doctorate degree = 2), marital status (Unmarried/divorced/widowed = 0, Married = 1), number of children needing care (None or zero = 0, At least one = 1), and job type [Doctor or nurse = 0, Center for disease control (CDC) staff = 1, Others = 2].

#### Response Variable

The response variable of mental health was comprised of three indicators: anxiety symptoms, depressive disorders, and post-traumatic stress disorder (PTSD). Anxious symptoms were assessed with the Chinese version of Generalized Anxiety Disorder questionnaire-7 (GAD-7), which is a self-report questionnaire with seven items scored on a scale of 0 to 3. The participants were asked if they had experienced any of the items included during the last 2 weeks. Each item was given a severity score of 0 (none), 1 (a few days), 2 (more than half the time), or 3 (almost every day). Scores could range from 0 to 21. A higher score represented a greater level of anxiety. The Chinese version of Patient Health Questionnaire-9 (PHQ-9) was used to assess depressive disorders; it included nine items scored from 0 to 3 (0 = not at all, 1 = several days, 2 = more than half the days, 3 = nearly every day). The total score could range from 0 to 27. A higher score represents more severe depressive disorders. The Chinese-version of GAD-7 and PHQ-9 were widely used in the epidemiological investigation and widely accepted as simple self-management tools for screening depression or anxiety symptoms with good validity and reliability in Chinese population (25, 26).

Post-traumatic stress disorder (PTSD) is a mental health condition that develops in reaction to a terrifying and traumatic event. This study used the Chinses version of Posttraumatic Stress Disorder Checklist for DSM-5 (PCL-5) scale to evaluate PTSD. The scale included 20 items and each item was rated on a 5-point Likert scale (from 0 = not at all to 4 = extreme). A higher score represents more severe posttraumatic stress disorders. Previous studies targeted at medical workers and using the Chinses version of PLC-5 showed the Cronbach's α coefficient ranged from 0.86 to 0.93 (27).

The Cronbach's α coefficients of PHQ-9, GAD-7 and PCL-5 in current study were 0.917, 0.939 and 0.971, respectively.

#### Explanatory Variables

The explanatory variables of this study included workload, social support and occupational protection. Workload contained three indicators: total work days employed in the fight against the COVID-19 outbreak, work hours per day (<10 = 0, 10~12 = 1, > = 12 = 2), and number of hours between breaks (<6 = 0, > = 6 = 1).

The self-constructed measurement of perceived social support was designed after discussions with professionals and conformed to the actual source of support of healthcare workers during COVID-19 outbreak. Participants were asked to evaluate the degree of perceived social support they received from seven sources: government department, work unit, epidemic prevention headquarters, counterpart support unit, friends, colleagues, and family members. For each source of support, the level of support was classified into four categories: none = 1, fair = 2, modest = 3, and high = 4. A total social support score was calculated by adding together the scores from each of the sources of support. A higher score represented a higher level of social support. This self-constructed measuring tool for perceived social support exhibited an excellent internal consistency with a Cronbach's α of 0.934.

To evaluate the degree of occupational protection, participants were asked whether or not they had access to a sufficient supply of ten types of protective equipment: medical surgical masks, medical protective masks, KN95/N95 or more protective particulate respirators, disposable surgical masks, goggles, well-rounded hood or protective face shield, positive pressure or respiratory protection device, protective gloves, protective clothing and medical protective clothing. Occupational protection was defined as a binary variable. It was regarded as 1 if all the 10 items of protective equipment were sufficient; otherwise, it was regarded as 0.

#### Mediating Variable

The mediating variable was fatigue. A simple self-designed scoring scale was used to obtain the level of perceived fatigue of healthcare workers. Participants were asked to evaluate the degree of their fatigue during the past week. A continuous scale ranged from 0 to 10 was used for the evaluation (0 = no fatigue, 10 = burn out).

### Statistical Analyses

Data archiving and statistical analysis were performed using SPSS version 17 and IBM SPSS Amos version 21. The level of significance was set at *p* < 0.05. Descriptive analysis was carried out for socio-demographics variables, workload, social support, fatigue, and mental health. Continuous variables are presented as the mean and standard deviation (SD), while categorical variables are presented as frequency and percentage. To establish the conditions necessary for the test of the mediation relationship (28), Pearson's correlation analysis was conducted to examine the correlations among continuous variables. One-way ANOVA test was used to test correlations between categorical variables and continuous variables.

**Figure 1:**
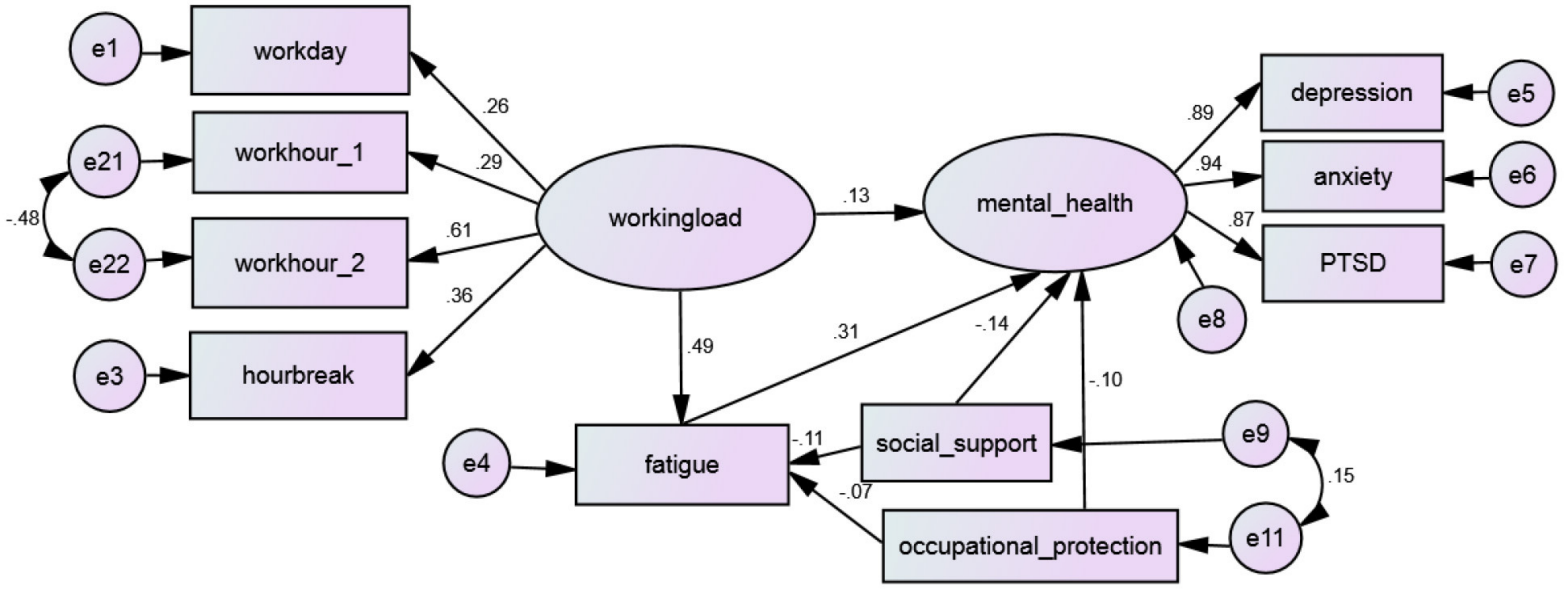
Structural equation modeling of fatigue as mediator of mental health.

Structural equation modeling (SEM) was conducted to test the mediating effect of fatigue on the relationship between the response variable (e.g., mental health) and explanatory variables (e.g., workload, social support, occupational protection). A graphic presentation of the SEM model appears in Figure 1. A fully mediated relationship occurs when the influence of the explanatory variable occurs through the mediator, whereas a partially mediated relationship occurs when the influence of the explanatory variable is transmitted both as a direct effect by and an indirect effect through the mediator variable (28).

Missing data were imputed by using a multiple imputation method and a fully conditional specification technique (29). A robustness test of mediating effect was conducted by bootstrapping with 2,000 random samples (30). The fit of the model was evaluated and confirmed by indices that included a chi-square estimate with degrees of freedom, normed chi-square (equal to chi-square divided by its degree of freedom, values < 5), the root mean square error of approximation (RMSEA, values < 0.08) and its 95% CI, the Tucker-Lewis index (TLI, values > 0.9), and the comparative fit index (CFI, values > 0.9).

## Results

### Description of the Study Sample

Demographic details of the participants are presented in Table 1. Most participants were females (63.4%), were married (74.2%), were doctors or nurses (70.6%), held at least a bachelor's degree (79.3%) and had at least one child needing care (65.7%). The mean age of participants was 37.1 years old.

**Table 1:** Descriptive statistics of survey data.

**Variables**	**Total (*N* = 2061)**
**Demographic variables**	
Age, mean ± SD	37.08 ± 9.08
**Gender (*****n*****, %)**	
Male	755 (36.6)
Female	1,306 (63.4)
**Education level (*****n*****, %)**	
Degree below bachelor	427 (20.7)
Bachelor's degree	1,218 (59.1)
Master's or doctorate degree	416 (20.2)
**Marital status (*****n*****, %)**	
Unmarried/divorced/widowed	532 (25.8)
Married	1,529 (74.2)
**Number of children needed care (*****n*****, %)**	
None or zero	707 (34.3)
At least one	1,354 (65.7)
**Job type (*****n*****, %)**	
Doctor or nurse	1,455 (70.6)
CDC staff	338 (16.4)
Others	268 (13.0)
**EXPLANATORY VARIABLES**	
**Workload**	
Total work days, mean ± SD	29.6 ± 9.90
**Work hours per day (*****n*****, %)**	
<10	1,388 (67.4)
10~	345 (16.7)
> = 12	328 (15.9)
**Continuous working hours (*****n*****, %)**	
<6	980 (47.5)
> = 6	1,081 (52.5)
Social support	5.81 ± 2.493
**Occupational protection**	
Sufficient	304 (14.8)
Not sufficient	1,757 (85.2)
**Mediating variable**	
Fatigue, mean ± SD	5.81 ± 2.494
**RESPONSE VARIABLES**	
**Mental health**	
Depressive disorders, mean ± SD	5.38 ± 5.098
Anxiety symptoms, mean ± SD	3.79 ± 4.236
Post-traumatic stress disorder, mean ± SD	10.25 ± 12.699

*SD, standard deviation; CDC, the centers for disease control*.

### Workload, Social Support, Occupational Protection, Fatigue and Mental Health

The mean total work days of the participants was approximately 30 days. The longest work days were 62 days. One-third of the participants worked shifts of 10 h or more. Most participants (52.5%) worked more than 6 h prior to their breaks. The majority (85.2%) of participants reported that they did not receive sufficient protective equipment. More than half of the participants reported moderate to high degree of fatigue (scores > = 5).

The scores of anxiety symptoms (mean ± SD = 3.79 ± 4.236), depressive disorders (mean ± SD = 5.38 ± 5.098) and PTSD (mean ± SD = 10.25 ± 12.699) ranged from 0–27, 0–21, and 0–80, respectively. When a cutoff value of 10 was taken (31, 32), 13.5 and 7.4% participants exhibited depressive disorders and anxiety symptoms, respectively. Only 5.7% of the participants exhibited PTSD, with morbidity defined as a score of 33 or above (33).

### Correlations Among Study Variables

Table 2 shows correlations among the study variables, including workload, social support, occupational protection, fatigue, and mental health. All correlations were significant. The biggest correlation coefficient and F-value were 0.398 and 157.409, respectively. All the workload indicators correlated positively with fatigue, anxiety, depression, and PTSD. However, both social support and occupational protection were negatively associated with fatigue and three mental health subscales.

**Table 2:** Correlation analysis results indicating correlations among explanatory variables, mediation variable and response variables.

	**Fatigue**	**Anxiety**	**Depression**	**PTSD**
Total work days	0.149^***^	0.105^***^	0.099^**^	0.058^**^
Work hours per day#	157.409^***^	48.786^***^	49.304^***^	37.230^***^
Continuous working hours#	89.641^***^	17.729^***^	23.791^***^	14.892^***^
Social support	−0.157^***^	−0.199^***^	−0.205^***^	−0.169^***^
Occupational protection#	35.944^***^	51.345^***^	55.176^***^	37.756^***^
Fatigue		0.370^***^	0.398^***^	0.325^***^

** *P < 0.01*,

*** *P < 0.001; # F-value of one-way ANOVA test was reported for categorical variables*.

### Mediating Effect of Fatigue

The fit indices of the structural equation models are presented in Table 3. The model fitting of the mediating effect of fatigue throughout the entire sample was satisfactory [χ^2^ (29, *N* = 2061) = 115.074, *p* < 0. 001; TLI = 0.978, CFI = 0.986, RMSEA = 0.038]. The *P*-value of all standardized path coefficients was <0.001, indicating that the relationships among explanatory variables, response variables, and the mediating variable were significant.

**Table 3:** Decomposition of mediating effects of fatigue for the structural model.

**Explanatory variables**	**Effect**	**Standardized coefficients**	**Percentage of SC**	**Lower bounds**	**Upper bounds**
Working load	Direct	0.126	45.818	0.053	0.201
	Indirect	0.149	54.182	0.123	0.179
	Total	0.275	100.00	0.210	0.341
Social support	Direct	−0.143	80.337	−0.196	−0.094
	Indirect	−0.035	19.663	−0.050	−0.021
	Total	−0.178	100.00	−0.233	−0.130
Occupational Protection	Direct	−0.096	76.641	−0.132	−0.056
	Indirect	−0.023	23.359	−0.038	−0.010
	Total	−0.119	100.00	−0.156	−0.077

*SC, standardized coefficients. All the coefficients are significant at level 0.001. The response variable is mental health*.

The decomposition of direct and indirect effects of each factor in the structural model also proved noteworthy. Table 3 illustrates the direct positive effect workload had on mental health. Additionally, social support and occupational protection were significantly directly and negatively correlated with mental health problems. Fatigue significantly mediated the indirect effects that all three explanatory variables had on mental health. For example, both the direct (β = 0.126, *p* < 0.001) and mediated effects (β = 0.148, *p* < 0.001) of workload on mental health were significant. Among the total effects (β = 0.275, *p* < 0.001), the direct effect accounted for 45.8% of the total effect and the indirect effect accounted for 54.2%. These findings indicated that fatigue partially mediated the relationship between workload, social support, occupational protection, and mental health problems.

Comparatively, the total effect of workload on mental health (β = 0.275, *p* < 0.001) was higher than that of both social support (β = −0.178, *p* < 0.001) and occupational protection (β = −0.119, *p* < 0.001). The proportion of indirect effect in the total effect of workload (54.2%) was higher than that of social support (19.7%) and occupational protection (23.4%) on mental health.

## Discussion

This study provided evidence that workload, social support, and occupational protection, as well as fatigue, had direct impacts on the mental health status of healthcare workers. The findings reaffirmed those from previous studies that identified connections between long work shifts, shortages of protective equipment, physical fatigue, and the absence of social support and psychological disorders among healthcare workers (15, 34). These workers could experience significant stress during major public health events like the COVID-19 pandemic. The stress experienced could contribute to anxiety symptoms, depression, or PTSD. However, social support and occupational protection proved to mitigate the impacts of those stressors on mental health. Excessive workload and adverse work conditions could exacerbate these impacts on mental health. Therefore, strategies to reduce the demands placed on these workers could alleviate the impact of these additional stressors.

The results of this study supported the hypothesis that fatigue represented a mediating variable in healthcare workers' mental health challenges. The findings included the direct and indirect (e.g., through fatigue) effects of three explanatory variables. When fatigue was controlled, the strength of the relationship between the explanatory variables and response (mental health) was reduced, indicating that fatigue partially mediated the effect of workload, social support, and occupational protection on mental health problems. Since several empirical studies had examined the effect of working load on mental health (35, 36) of nurses and physicians, the knowledge of the mechanism is limited. From a theoretical point of view, workload may affect mental health because of potential direct impact of working hours on leisure or the time available for health production at home (37). Moreover, workload may affect mental health because of potential indirect impact of working burden on the job, such as physically strenuous work leading to exhaustion (physical fatigue), and psychologically demanding work leading to stress (mental fatigue) (38, 39). During the peak of the COVID-19 pandemic in China, the job of healthcare workers were characterized by long working hours and great psychological stress (7, 9). Consequently, this study revealed the link between working load and mental health via a mediator of fatigue by using a nation-wide survey target at Chinese healthcare workers fought against the COVID-19.

In terms of relationship among social support, fatigue and mental health, previous studies reported that social support had negative correlation with fatigue symptoms in healthcare workers (40) and protected against depression or psychological health problems (41). Literature indicated that fatigue may have mediating effect in the relationship between social support and mental health (42). This study provided an empirical evidence to support the view of the literature mentioned above.

In addition, occupational exposure was regarded as a risk factor associated with psychological state especially in the face of major communicable diseases (43). In theory, lack of sufficient protection may lead to psychological panic such as fear of infection, reduced activity and motivation, physical fatigue, which may further affect people's mental health. By using unique empirical data, this study revealed the possible mediating effect of fatigue on the relationship between mental health and occupational protection.

The findings may have practical implications for the design of effective mental health interventions for healthcare workers during second wave of the COVID-19 pandemic. First, the results highlighted the importance of targeting fatigue. Because fatigue can manifest as both mental and physical fatigue, interventions should be designed to address both. Second, the direct effect of workload on mental health was lower than its indirect effect mediated by fatigue. However, the direct effect of social support as well as occupational protection on mental health was much higher than the indirect effect mediated by fatigue. This finding indicated that inventions designed to address mental health problems should emphasize the reduction of fatigue for healthcare workers who carry heavy workloads. Third, the addition of fatigue as a mediating variable in this study did not cancel out the direct effect of explanatory variables on mental health problems. This meant that workload, social support, and occupational protection also could contribute to healthcare workers' mental health outcomes. Therefore, a reduction in workload and improved access to social support and sufficient protection equipment would contribute to their improved mental health during events such as a COVID-19 outbreak. It was suggested that Telemedicine and e-Health Systems should be used to save time and to improve working efficiency. Healthcare institutions and organizations should take into account social support such as informational support, appraisal support, instrumental support, and emotional support in their day-to-day operations. Healthcare workers would benefit from availability of personal protection equipment as well as equipment training.

There are several limitations to this study. Although the findings provided a basis for a better understanding of the relationships among the variables studied, they could be further validated in experimental and longitudinal studies. Moreover, fatigue was measured by using only one self-rated question in this study. Therefore, there was no distinction between mental and physical fatigue. However, to some extent, self-reported fatigue scores could directly reflect the degree of fatigue of the participants. In addition, this way of reporting fatigue scores was reasonable since our survey was conducted at a special point in time. The survey period corresponded to a period of decline following the highest rate of infection during the COVID-19 epidemic outbreak in China. Since most of the participants were still burden by the task of controlling the epidemic, a brief evaluation of fatigue would be time-saving and improved the response rate of this survey. A comprehensive scale of fatigue should be included in future research on this topic. Finally, future studies could examine other factors or mechanisms (such as stress, burnout, and job satisfaction) that could mediate the relationship between the explanatory variables and mental health problems to help improve understandings of appropriate interventions for healthcare workers who experience mental health challenges.

## Conclusions

This was the first study to investigate the mediating role of fatigue in the relationship between mental health problems and its multiple risk factors by using a large sample of healthcare workers engaged in the fight against COVID-19 in China. The findings indicated that fatigue was an important mediator of the relationship between workload, social support, and occupational protection and mental health. The results highlighted the importance of potential interventions to alleviate fatigue and consequently improve the mental health status of healthcare workers.

## Data Availability Statement

The raw data supporting the conclusions of this article will be made available by the authors, without undue reservation.

## Ethics Statement

The study protocol was approved by the Research Ethics Committee of Sun Yat-sen University, Guangzhou, China. Because the questionnaire was anonymous, we assumed that participants consented to participate in our study by returning the questionnaire.

## Author Contributions

RP and LL designed the study. DZ, MC, and WZ collected the data. RP, DZ, MC, and WZ analyzed the results. RP wrote the main manuscript text. LL, DZ, MC, and WZ reviewed the manuscript. All authors contributed to the article and approved the submitted version.

## Conflict of Interest

The authors declare that the research was conducted in the absence of any commercial or financial relationships that could be construed as a potential conflict of interest.
